# Severe Scratching in Spinocerebellar Ataxia 17: Another Case

**DOI:** 10.5334/tohm.235

**Published:** 2020-07-07

**Authors:** Martje G. Pauly, Alexander Münchau, Norbert Brüggemann

**Affiliations:** 1 Institute of Neurogenetics, University of Lübeck, Lübeck, DE; 2 Department of Neurology, University Hospital Schleswig Holstein, Lübeck, DE

**Keywords:** SCA17, scratching, self-injurious behavior, chorea, ataxia

Dear Editor,

With great interest, we read the case report “Self-injurious behaviour in SCA17: a new clinical observation”[1] by Bonomo and colleagues, describing two patients with spinocerebellar ataxia type 17 (SCA17) who presented with repetitive skin scratching. While self-injurious behavior is a common feature in mental retardation [2] and primarily psychiatric disorders [3], this was the first report of this phenomenon in patients with SCA17.

We here report another patient with SCA17 presenting with severe skin scratching.

Initially, this 72-year old patient with a positive family history noticed slurred speech and progressive gait impairment, including balance problems around the age of 56. The disease was then characterized by progressive cognitive decline, moderate generalized chorea, dysarthria, and limb and gait ataxia (SARA 12/2018: 16/40, 09/2019: 21.5/40, MoCA 05/2016: 11/30). Genetic testing in 2011 revealed 38 and 50 CAG repeats in the *TBP* gene (cutoff 48 repeats), confirming the diagnosis of SCA17. An MRI in 2014 showed global brain atrophy including cerebellar volume loss with a predominant involvement of the vermis. An individualized treatment with the antiglutamatergic drug riluzole was initiated in 2017 to treat ataxia symptoms. In 2018, the wife reported an increase of disorientation and restlessness, which progressed over the following months. A therapy with quetiapine was started (initially 50mg daily, later up to 100mg daily), which did not significantly improve restlessness. In June 2019, two years after the initiation of riluzole therapy, the patient started to severely scratch himself mainly at the neck, trunk, and arms. Due to progressive dementia, he was not able to provide meaningful information regarding scratching, particularly the presence of itchiness or an urge to scratch without the sensation of itchiness; the wife, however, confirmed a possible relation to the increase of general restlessness (Figure 1 A, B). Therapy with antihistamines only led to a slight improvement. A brief interruption of riluzole therapy did not alter the scratching. A skin biopsy showed no signs of an autoimmune dermatosis, particularly no evidence for bullous pemphigoid.

**Figure 1:**
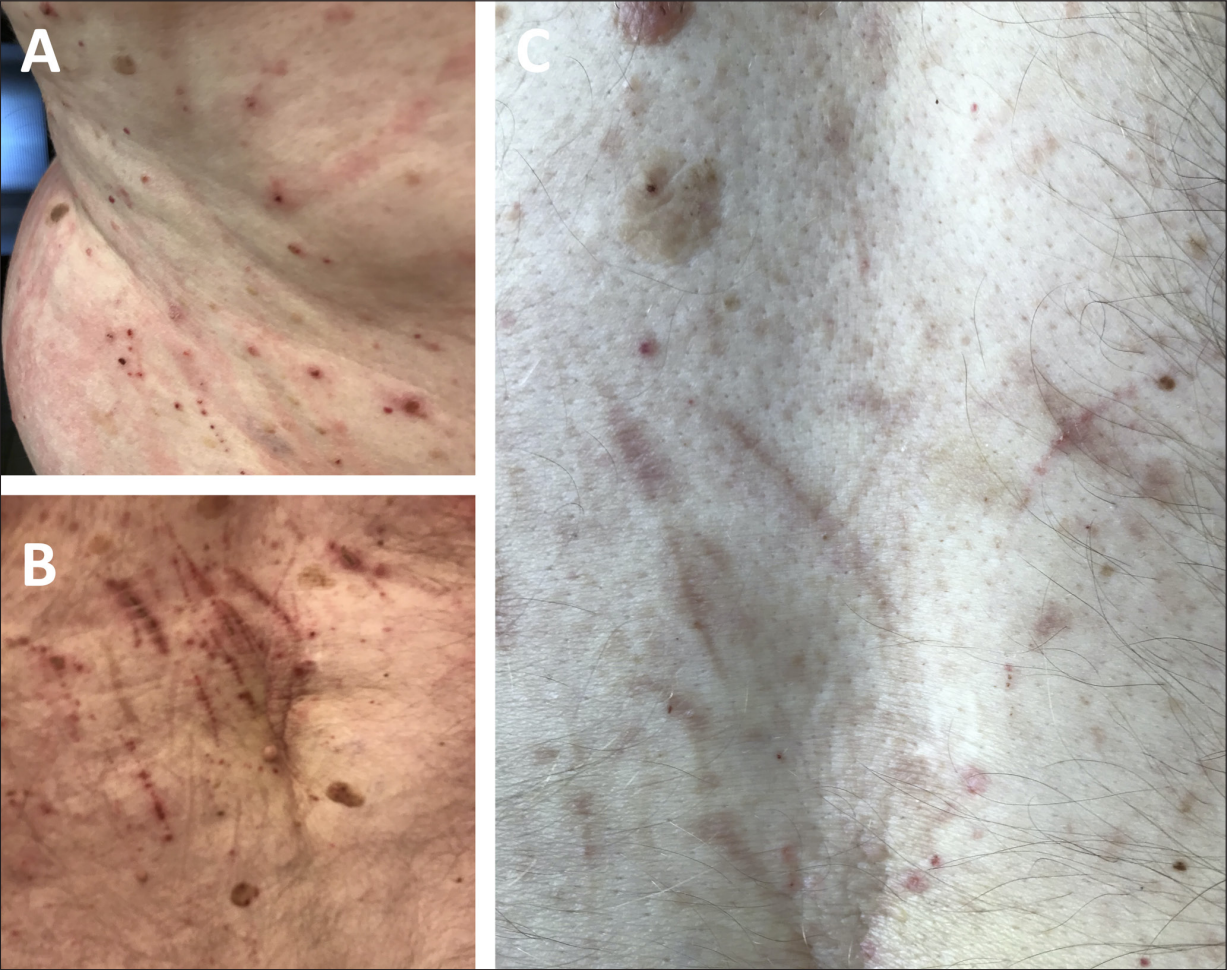
**Photographs of skin lesions.** Figure legend: In August 2019 (A, B) and after (C) the change of medication in October 2019. **(A)** Lateral trunk, **(B)** lower back, **(C)** lower back.

Also, there was no evidence for an underlying hepatic disease (e. g. as a side effect of riluzole) causing the pruritus and no obvious association with a change in medication. A functional cause of the scratching was suspected. A change of medication to risperidone with an optional addition of melperone was suggested.

Before the change in medication was implemented and following an acute psychotic exacerbation with agitated behavior, the patient was admitted to an external gerontopsychiatric facility. Riluzole was stopped, and therapy with risperidone was initiated. Under the new medication with risperidone and trazodone scratching markedly improved (Figure 1C). Due to rapid cognitive decline, care at home was no longer feasible, and the patient was referred to a home for people with neuropsychiatric disorders, where he deceased a couple of weeks later at the age of 72 years due to an unknown cause.

Interestingly, our patient, as well as both previously reported cases, presented with chorea confirming SCA17 to be an important differential diagnosis of Huntington’s disease [4], in which itching is reported to be relatively frequent [5]. Our case highlights that disinhibition and general restlessness should be considered as a possible cause of clinically troublesome scratching in patients with SCA17 and possibly also other neuropsychiatric diseases, provided potentially other underlying causes including skin and liver diseases have been excluded. Pharmacological management in these cases may include high potency neuroleptics. Neurologists should thus be aware of this potentially under-recognized clinical sign in neurodegenerative disorders with a broad spectrum of neuropsychiatric symptoms.

## Author Information

MGP: The author is supported by the section of medicine of the University of Lübeck (CS06-2020). She is a member of the European Reference Network for Rare Neurological Diseases – Project ID No 739510.

AM: Commercial research support: Pharm Allergan, Ipsen, Merz Pharmaceuticals, Actelion; Honoraria for lectures: Actelion, GlaxoSmithKline, Desitin and Teva; Support from Foundations: Possehl-Stiftung (Lübeck, Germany), Margot und Jürgen Wessel Stiftung (Lübeck, Germany), Tourette Syndrome Association (Germany), Interessenverband Tourette Syndrom (Germany), CHDI; Academic research support: Deutsche Forschungsgemeinschaft (DFG): projects 1692/3-1, 4-1, SFB 936, and FOR 2698 (project numbers 396914663, 396577296, 396474989), Innovationsausschuss of the Gemeinsamer Bundesausschuss: Translate NAMSE (structural support for the Lübeck Center for Rare Diseases); Royalties for the book Neurogenetics (Oxford University Press). AM is a member of the European Reference Network for Rare Neurological Diseases – Project ID No 739510.

NB: The author received speaker’s honoraria from Grünenthal, UCB, Abbvie and Teva. He served as a consultant for Censa Pharmaceuticals and Centogene. He received non-financial support from Bayer. NB is funded by the DFG (BR4328.2-1, GRK1957), the Collaborative Center for X-linked Dystonia-Parkinsonism and the Else-Kröner Fresenius-Stiftung (HA17_2017).
